# Glycemic and Insulin Status in Periodontitis Patients using the Homeostasis Model Assessment (HOMA): A Pilot Study

**DOI:** 10.3290/j.ohpd.b3818027

**Published:** 2023-01-18

**Authors:** Elisabeth Gerin, Martin Buysschaert, Jérôme F. Lasserre, Julian G. Leprince, Selena Toma

**Affiliations:** a Periodontist, Department of Periodontology, University Clinics Saint-Luc, Catholic University of Louvain (UCLouvain), Brussels, Belgium. Idea, hypothesis, experimental design, performed the experiments in partial fulfillment of requirements for a degree, data collection, wrote the manuscript.; b Professor Emeritus, Department of Endocrinology and Nutrition, University Clinics Saint-Luc, Catholic University of Louvain (UCLouvain), Brussels, Belgium. Hypothesis, experimental design, contributed substantially to discussion, proofread the manuscript.; c Periodontist and Lecturer, Department of Periodontology, University Clinics Saint-Luc, Catholic University of Louvain (UCLouvain), Brussels, Belgium Idea, hypothesis, experimental design, contributed substantially to discussion, proofread the manuscript.; d Professor, Department of Adult and Child Dentistry, Cliniques Universitaires Saint-Luc, Brussels, Belgium. DRIM Research Group & Advanced Drug Delivery and Biomaterials, Louvain Drug Research Institute, UCLouvain, Brussels, Belgium. Contributed substantially to discussion, proofread the manuscript.; e Associate professor Department of Periodontology, University Clinics Saint-Luc, Catholic University of Louvain (UCLouvain), Brussels, Belgium. Idea, hypothesis, experimental design, contributed substantially to discussion, proofread the manuscript.

**Keywords:** diabetes, periodontal disease, risk factor(s), systemic health/disease

## Abstract

**Purpose:**

This study aimed to compare insulin status and dysglycemia (prediabetes/diabetes) of patients with chronic (stage III, grade B) or aggressive periodontitis (stage III, grade C) to that of a healthy population.

**Materials and Methods:**

Patients with chronic (CP, n = 16) or aggressive periodontitis (AP, n = 15) and periodontally healthy controls (n = 32) were recruited. Body mass index was calculated. Glycemia, plasma insulin, glycated hemoglobin, C-reactive protein, and lipid levels were measured in fasting. The Homeostasis Model Assessment was used to calculate the insulin sensitivity (HOMA-%S), the beta-cell function (HOMA-%B), and their hyperbolic product (HOMA-%BxS).

**Results:**

The CP group showed statistically significantly insulin resistance with a lower HOMA-%S (p = 0.0003) and a reduced HOMA-%BxS (p = 0.049) despite a higher insulin level (p = 0.01) vs the control group, even after BMI adjustment. There was also a trend to dysglycemia (prediabetes/diabetes) in the chronic group. In patients with AP, no abnormalities in insulin status were observed and glycemic levels were comparable with controls. Additionally, patients in both AP and CP groups presented significantly higher CRP levels compared to those of the control group (p = 0.02).

**Conclusion:**

Patients with CP showed reduced insulin sensitivity, increased insulin levels but a reduced %BxS product and a trend to dysglycemia. These abnormalities were not observed in AP.

Diabetes mellitus and periodontitis are two disorders ranked among the most prevalent in the global burden of disease, and contribute to major health expenditures. Dental diseases and diabetes are ranked first and third, respectively, at the European Union level.^43^

Diabetes mellitus is a pandemic metabolic disease found worldwide, with 463 million adults diagnosed in 2019.^18^ The International Diabetes Federation further reported that 4.2 million deaths can be attributed to this disorder.^18^ In addition to those with conventional forms of diabetes, 374 million people exhibit a glucose level too high to be considered normal, but too low for them to be diagnosed with diabetes. This condition is referred to as “prediabetes” or “intermediate hyperglycemia” (impaired fasting glycemia/impaired glucose tolerance), and is a major risk factor for future development of diabetes and cardiovascular complications.^1,7^

Periodontitis is a multifactorial inflammatory disease associated with dysbiotic plaque biofilms characterised by progressive destruction of the tooth-supporting apparatus.^32^ Chronic periodontitis (CP) is the most frequent form of the disease, with relatively slow progression, while the aggressive (AP) form has rapid and highly destructive effects.^2^ Periodontal disease (AP and CP) has been associated with increased levels of inflammatory biomarkers, including C-reactive protein (CRP).^15,39^ In June 2018, during data collection for this study, a new classification for periodontitis was introduced. The terms “aggressive” and “chronic” periodontitis were removed. Periodontitis is classified by stages, depending on the severity, and by grades, depending on the progression rates. AP and its rapid destruction (equivalent of stage III, grade C) and CP and its slower progression (equivalent of stage III, grade B) are still reflected in the grades.^32^

It has been suggested that (pre)diabetes and periodontitis have a two-way causal relationship.^17,20,30,36^ However, while long-term hyperglycemia has been associated with a decrease in the diversity of the oral microbiome^34,41^ and has been identified as one of the main risk factors for chronic periodontitis,^14,30^ the influence of periodontal disease (chronic or aggressive) on the development of prediabetes or diabetes is still unclear. Some reports have indeed indicated an increased risk of insulin resistance,^40^ dysglycemia,^8,37^ and/or a poorer long-term glycemic control in diabetic patients,^18,38^ while others have not confirmed such disease-related associations.^19,20^ One study also connected diabetes and periodontitis through endotoxemia,^36^ but none of these studies have looked deeper into the Beta-cell function of the pancreas underlying development of (pre)diabetes. Moreover, no reports differentiated potential metabolic abnormalities between CP and AP.

Beta-cell function in the pancreas can be estimated using homeostatic model assessment (HOMA), which is a mathematical model that uses the insulin and fasting plasma glucose levels to determine insulin sensitivity (%S) and steady-state pancreatic beta-cell function (%B). Multiplying %B by %S (hereafter denoted %BxS) yields a hyperbolic curve describing the relationship of relative beta-cell function to insulin sensitivity. Normal beta-cell function is represented by a %BxS value of 100%.^26^

Insulin sensitivity and beta-cell function regulate insulin secretion via a feedback loop, which aims to maintain glucose tolerance. If there is a decrease in insulin sensitivity, pancreatic beta cells will compensate by increasing their activity; conversely, beta cells lower their activity if insulin sensitivity is high. In both cases, %BxS remains within the normal range. If this feedback loop does not work effectively, %BxS decreases, leading to prediabetes, which in turn can lead to diabetes.

Building on this research, the aim of the current pilot study was to screen patients diagnosed with chronic or aggressive periodontitis for dysglycemia (prediabetes/diabetes) on the hypothesis that CP and AP will show differences in terms of their insulin sensitivity and secretion patterns.

## Material and Methods

### Ethics Approval

This study and its protocol were approved by the ethics committee of the local institution (Clinical Trial Center of the University Clinics Saint-Luc, Brussels, Belgium; approval no. 2017/22DEC/571) and all participants signed an informed consent form. The study was conducted in accordance with the guidelines of Good Clinical Practice and the revised Declaration of Helsinki for clinical studies. This study used the STROBE checklist as a guideline.

### Patient Selection

Patients were prospectively selected from individuals attending the School of Dental Medicine and Stomatology at the University Clinics Saint-Luc (Université catholique de Louvain) between April 4th, 2018 and June 10th, 2019. Patients with periodontal disease were included in two different groups using the definitions of periodontitis in the classification scheme developed by Armitage et al,^2^ i.e. aggressive periodontitis and severe generalised chronic periodontitis. The diagnoses were made based on a periodontal examination (periodontal pocket depth, bleeding on probing, clinical attachment loss), always done by the same examiner. The patients in the AP group (corresponding to stage III, grade C in the new classification) were 18–35 years old, had no pre-existing medical conditions that could explain the periodontal lesions, and showed aggressive periodontal destruction on at least the first molar and/or the incisor. Additionally, they had not received periodontal treatment in the six months preceding the screening. The patients in the CP group showed clinical signs of severe generalised periodontitis, corresponding to stage III, grade B in the new classification, being defined as a periodontal pocket depth (PPD) of 6 mm affecting >30% of probing sites. Additionally, to improve the discrimination between the patients of the two groups, those included in the CP group were ≥40 years old. In both groups, only patients with no history of periodontal treatment in the last 6 months were included.

The control group was selected from the students, dentists, and employees of the School of Dental Medicine and Stomatology at the university hospital. To be included, control subjects were required to have a Dutch Periodontal Screening Index (DPSI) ≤2, no prior history of periodontal disease, and be in good general health. The subjects in the control group were age-matched to those in the periodontitis groups according to the following ranges (in years of age): <20; 20–24; 25–29; 30–34; 35–39; 40–45; and >50.

The characteristics of the selected individuals and their smoking status can be observed in Table 1.

**Table 1:** Characteristics of the study participants and prevalence of dysglycemia

	Patients with periodontitis		Control subjects
	Aggressive	Chronic	
n (patients)	16	15	32
Sex (M/F), %	38/62	60/40	47/53
Age (years), mean ± SD	28 ± 5	49 ± 6	39 ± 13
BMI (kg/m^2^), mean ± SD	26 ± 3	27 ± 4	24 ± 3
Current smoker (%)	25	20	3
Prediabetes (%)	19	47	34
Diabetes (%)	0	13	0
Prediabetes/diabetes^1^ (%)	15	60	34

^1^Chronic vs control and aggressive, p = 0.11. Aggressive periodontitis = stage III, grade C; chronic periodontitis = stage III, grade B.

### Data Collection

Body mass index (BMI; defined as weight/height^2^) was calculated for each patient and control subject.

Prior any kind of periodontal treatment, subjects were asked to have a blood test according to the prescription we had given to them. The blood sample was done at the University Clinic Saint-Luc of Brussels by the staff of the Collection Center. The blood test was performed on the individuals in the three groups during fasting. The following parameters were measured: plasma glucose (mg/dl); plasma insulin (pmol/l); glycated hemoglobin (HbA1c; %); high-sensitivity CRP (mg/l); triglycerides (mg/dl); total cholesterol (mg/dl); high-density lipoprotein and low-density lipoprotein cholesterol (HDL/LDL; mg/dl).

Prediabetes and diabetes were assessed based on the conventional American Diabetes Association (ADA) criteria (prediabetes: fasting plasma glucose [FPG] ≥100–125 mg/dl, and HbA1c, 5.7–6.4%; diabetes: FPG ≥126 mg/dl, and HbA1c ≥6.5%) (ADA 2020). Insulin sensitivity (HOMA-%S), beta-cell function (HOMA-%B), and their hyperbolic product (HOMA-%BxS) were calculated using HOMA. The HOMA calculator can be downloaded from the website of the Diabetes Trial Unit of the University of Oxford, https://www.dtu.ox.ac.uk/homacalculator/download.php.^26^ We considered abnormal HOMA-%B and/or HOMA-%S to correspond to values <70% of the normal value.

### Statistical Analysis

Continuous variables are presented as the mean ± SD, and categorical variables as numbers and proportions. Data distribution was assessed for normality using the Shapiro-Wilk test; non-parametric tests were used where appropriate. For the fitted normal distributions, one-way ANOVA was applied to glycaemia, HbA1C, cholesterol, HDL, LDL and BMI. When the Shapiro-Wilk test did not detect a normal distribution, the Wilcoxon/Kruskall-Wallis test was applied to CRP, insulin, and HOMA values. A multivariate model including insulin sensitivity, subject age, and BMI was built using logistic regression. All statistical analyses were conducted with JMP software PRO 14 (SAS Institute; Cary, NC, USA). Last, a Χ^2^/Fisher test was conducted to compare the number of subjects with prediabtes/diabetes and HOMA under 70%. Statistical significance was set at p < 0.05.

## Results

Thirty-four patients (AP group: n = 17; CP group: n = 17) and 36 controls were included in the study. Of these 70 subjects, 63 had a blood sample taken; the seven who did not were subsequently excluded from the study (AP: n = 1; CP n = 2; controls: n = 6). After these exclusions, a total of 16 patients remained in the aggressive periodontitis, 15 in the chronic periodontitis, and 32 subjects in the control group (Table 1).

The demographic and clinical characteristics of the subjects in the three groups are indicated in Table 1. As expected, individuals with AP were younger than the subjects in the other groups. BMI were comparable in CP and AP patients.

**Fig 1:**
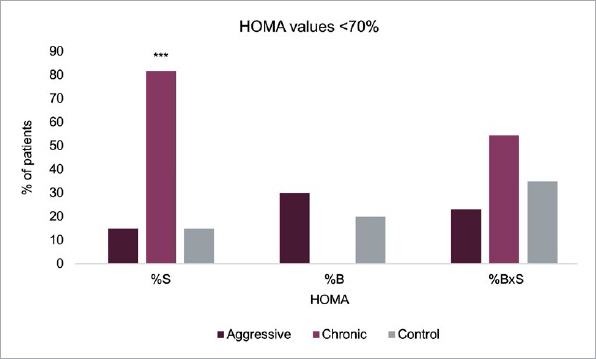
Comparison of the percentage of subjects with HOMA-%S, HOMA-%B, and HOMA-%BxS <70% in each group. ***p=0.0003.

As shown in Table 2, mean FPG and HbA1c levels were slightly (non-significantly) higher in patients with CP. Dysglycemia (prediabetes/diabetes) tended to be more frequent in the latter group (vs AP and controls), but the difference was not statistically significant (p = 0.11) (Table 1). Two patients with CP had type-2 diabetes. Plasma insulin levels were statistically significantly greater in subjects with CP vs controls (90 ± 45 vs 52 ± 31 pmol/l, p = 0.011). Insulin sensitivity (HOMA-%S) was statistically significantly lower in the CP group compared with the AP group (p = 0.007) and with controls (p = 0.0068), even after adjusting for BMI. Consistent with this, 82% of patients with CP showed insulin resistance, with HOMA-%S values <70% (vs 15% in the AP group and 15% in controls, p = 0.003) (Fig 1). As expected, HOMA-%B was higher in subjects with CP vs the other groups (p = 0.08). The hyperbolic product HOMA-%BxS was 70% ± 37% in the CP group, compared with 94% ± 26% (p = 0.02) and 95% ± 36% (p = 0.049) in the AP and control groups, respectively. In contrast, HOMA-%S, HOMA-%B, and HOMA-%BxS values were comparable in the AP and control groups. Compared to the control group (1.5 ± 1.0 mg/l), levels of CRP were statistically significantly higher in both the aggressive group (5.1 ± 6.4 mg/l, p = 0.008) and the chronic group (3.5 ± 4.7 mg/l, p = 0.02). In terms of cholesterol levels, those of HDL, LDL, and triglycerides did not differ statistically significantly between groups (Table 3).

**Table 2:** Laboratory markers

Parameters (mean ± SD)	Standards	Aggressive periodontitis	Chronic periodontitis	Control
CRP (mg/l)^1^	<5	5.1 ± 6.4	3.5 ± 4.7	1.5 ± 1.0
Fasting glycemia (mg/dl)	70–100	91.8 ± 9.2	99.1 ± 12.2	93.4 ± 9.1
HbA1c (%)	4.0–5.6	5.4 ± 0.4	5.5 ± 0.6	5.4 ± 0.3
Insulin (pmol/l)^2^	<130	60.0 ± 12.3	100.4 ± 66.4	50.1 ± 30.3
HOMA-S (%)^3^	100	107.5 ± 12.3	58.9 ± 32.1	108.4 ± 54.5
HOMA-B (%)	100	98.1 ± 9.8	129.0 ± 35.7	106.8 ± 60.1
HOMA-BxS (%)^4^	100	94.0 ± 26.5	70.3 ± 37.6	92.3 ± 36.3

^1^ Aggressive vs control, p = 0.008; chronic vs control, p = 0.04; ^2^chronic vs control, p = 0.01; ^3^chronic vs control p = 0.007; chronic vs aggressive, p = 0.008; ^4^chronic vs control, p = 0.049.

**Table 3:** Laboratory markers

Parameters (mean ± SD)	Standards	Aggressive periodontitis	Chronic periodontitis	Control
Cholesterol (mg/dl)	<190	165 ± 38.3	186.2 ± 31.1	181.5 ± 40.2
LDL (mg/dl)	<115	94.9 ± 34.3	111.8 ± 41.3	101.8 ± 33.9
HDL (mg/dl)	>40	54.7 ± 8.2	58.9 ± 12.5	58.7 ± 18.5
Triglycerides (mg/dl)	<150	76.6 ± 29.6	88.2 ± 38.2	97.2 ± 76.7

LDL: low-density lipid cholesterol; HDL: high-density lipid cholesterol; SD: standard deviation.

## Discussion

Our results suggest that the metabolic (insulin) patterns differ in these two forms of periodontal disease, with CP showing some statistically significant results.

Using HOMA tests, we demonstrated that CP is associated with a 50% reduction in insulin sensitivity, partially compensatory hyperinsulinemia, and a decreased %BxS vs controls, even after adjustment of BMI. This abnormalities were not present in subjects with AP. The latter difference could be explained in part by age^18^ as well as by the more rapid evolution of AP^24^ vs the slow development of (undiagnosed) CP^13^ that allows more time for metabolic disorders to evolve.^27^ Such a dysfunction in CP patients could lead over time to glucose disorders per se (prediabetes, diabetes), as has also been suggested by others.^24,29^

Along these lines, the degree to which dysglycemia is present in subjects with CP remains rather controversial.^9^ Arora et al^9^ and many other authors have reported an increased risk of prediabetes (impaired fasting glucose, impaired glucose tolerance) and diabetes accompanying severe periodontitis, but not mild and moderate CP.^4,9,10,14,22,44^ However, other data, in particular those from the Pomerania study (SHIP-TREND),^23^ have not confirmed such an association.^19,21,23^ Our data support these results, showing that insulin resistance and altered BxS% were already observed before the onset of glycemic disorders, as only a trend for more frequent dysglycemia was observed in CP individuals.

As expected in view of HOMA data, there was no increase in the prevalence of dysglycemia in patients with AP when compared with controls. These results are in line with previous studies, in particular that by Davies et al,^11^ who found no relationship between AP, dysglycemia, or altered levels of inflammatory biomarkers. Nibali et al^31^ observed an increase in non-fasting plasma glucose and inflammatory markers in severe periodontitis, including AP and generalised CP. However, they did not report any differences in the levels of metabolic or inflammatory parameters between AP and generalised severe chronic periodontitis.^31^

One limitation of our study is the rather small sample size. In addition, the systematic use of an oral glucose-tolerance test during screening would probably have increased our estimate of the number of patients with dysglycemia, as recently noted by Bergman et al.^5^ During data collection for this study, the periodontitis classification changed and the terms “chronic” and “aggressive” were removed. Periodontitis is currently classified as stages and grades. However, it might be considered that the terms “aggressive” or “chronic” already reflected the grade as a progression rate.

From a physiopathological point of view, progressive insulin and glycemic dysfunction with insulin resistance and subsequent prediabetes in severe forms of CP could be the consequence of chronically increased levels of proinflammatory cytokines, as reported elsewhere.^24,42^ Demmer et al^12^ proposed the alternative hypothesis that metabolic disorders could be related to the dysbiotic microbial communities found in periodontitis. Higher levels of colonisation by specific bacteria were associated with an elevated prevalence of prediabetes, increasing its prevalence by two- to three-fold.

In agreement with other authors,^28,34^ we found significantly higher levels of CRP in the CP and AP groups was comparable to that of the control group. CRP is a marker of acute or systemic inflammation, which is released in response to different cytokines (such as IL-6, IL-1, and TNF-α) associated with periodontitis.^6^

Our data have important clinical implications, as they could help periodontologists to cooperate with diabetologists to prevent (pre)diabetes, as already suggested by Sanz et al.^38^ In view of our results, it seems essential to screen for insulin resistance in CP patients, even in the presence of “still normal” plasma glucose levels, as an early prevention measure. Targeting and treating insulin resistance, in particular in these high-risk individuals, will indeed prevent the later development of glycemic disorders with their specific complications. Morever, as reported by several authors, insulin resistance by itself is also considered to be a high risk factor for CP, as reported in studies of obese normoglycemic individuals.^29^

## Conclusion

In this studied population, patients with CP (but not with AP) demonstrated insulin resistance and altered insulin function, as shown by the HOMA hyperbolic product. These abnormalities may be associated with the subsequent development of (pre)diabetes. Therefore, this study highlights the need for systematic metabolic (insulin-resistance) and glycemic screening in patients with CP. Further research is warranted to corroborate our findings.
